# MANAGEMENT OF *CLOSTRIDIOIDES DIFFICILE* INFECTION IN INFLAMMATORY BOWEL DISEASE: A SYSTEMATIC REVIEW AND META-ANALYSIS OF ANTIBIOTIC EFFICACY

**DOI:** 10.1590/S0004-2803.24612025-145

**Published:** 2026-07-24

**Authors:** Isadora Brandão PELUCIO, Carla MALAGUTI, Túlio Medina Dutra de OLIVEIRA, Karoline Maria de Souza Marques CHITARRA, Maria Cristina Vasconcellos FURTADO, Júlio Maria Fonseca CHEBLI

**Affiliations:** 1 Universidade Federal de Juiz de Fora (UFJF), Programa de Pós-Graduação em Saúde, Juiz de Fora, MG, Brasil.; 2 Universidade Federal de Juiz de Fora (UFJF), Departamento de Fisioterapia do Idoso, Adulto e Materno/Infantil, Juiz de Fora, MG, Brasil.; 3 Universidade Federal de Juiz de Fora (UFJF), Faculdade de Medicina, Programa de Pós-Graduação em Saúde, Juiz de Fora, MG, Brasil.; 4 Universidade Federal de Juiz de Fora (UFJF), Faculdade de Medicina, Curso de Medicina, Juiz de Fora, MG, Brasil.; 5 Universidade Federal de Juiz de Fora (UFJF), Faculdade de Medicina, Departamento de Cirurgia, Juiz de Fora, MG, Brasil.; 6 Universidade Federal de Juiz de Fora (UFJF), Faculdade de Medicina, Departamento de Clínica Médica, Juiz de Fora, MG, Brasil.

**Keywords:** Inflammatory bowel diseases, clostridioides difficile, anti-bacterial agents, systematic review, treatment outcome, Doença inflamatória intestinal, clostridioides difficile, antibióticos, revisão sistemática

## Abstract

**Background:**

*Clostridioides difficile* infection (CDI) is a significant complication in patients with inflammatory bowel disease (IBD), often associated with higher recurrence rates, increased morbidity, and complex therapeutic decision-making. The optimal antibiotic regimen for CDI in IBD remains uncertain.

**Objective:**

To evaluate the efficacy of different antibiotic therapies for CDI in patients with IBD.

**Methods:**

A systematic review and meta-analysis were conducted according to PRISMA 2020 guidelines. Searches were performed in MEDLINE, EMBASE, PubMed, Epistemonikos, and the Cochrane Library through October 2024. Observational studies and clinical trials comparing antibiotic regimens in adult IBD patients with CDI were included. Data on recurrence, clinical outcomes, colectomy, and mortality were extracted. Random-effects meta-analysis was performed when appropriate.

**Results:**

Nine studies involving 643 patients met inclusion criteria. Recurrence occurred in 17.4% of patients treated with metronidazole and 8.2% of those treated with vancomycin. In the meta-analysis of four studies (n=214), vancomycin showed a trend toward lower recurrence, although not statistically significant (OR 0.55; 95%CI 0.15-1.99; *P*=0.36; I^2^=66%). Mortality was low and similar between groups, and colectomy occurred exclusively in patients with ulcerative colitis. Data on clinical success and adverse events were heterogeneously reported and summarized narratively.

**Conclusion:**

Vancomycin may be associated with lower recurrence in higher-risk IBD patients; however, available evidence remains limited and heterogeneous. Treatment decisions should be individualized, and further prospective, IBD-specific studies are needed to clarify optimal management strategies.

## HIGHLIGHTS

*Clostridioides difficile* infection is a frequent complication in patients with inflammatory bowel disease.

Recurrence rates were higher among patients treated with metronidazole compared with vancomycin.

Meta-analysis showed a trend toward lower recurrence with vancomycin, although not statistically significant.

Evidence specific to IBD remains limited and heterogeneous.

Further prospective studies are needed to guide optimal treatment strategies.

## RESUMO

**Contexto:**

A infecção por Clostridioides difficile (ICD) é uma complicação relevante em pacientes com doença inflamatória intestinal (DII), associada a piores desfechos clínicos. Evidências sobre a eficácia comparativa de antibióticos nesse subgrupo ainda são limitadas.

**Métodos:**

A revisão sistemática foi conduzida nas bases MEDLINE, EMBASE, PubMed, Cochrane Library e Epistemonikos, incluindo estudos observacionais publicados até outubro de 2024. Foram incluídos pacientes com DII e ICD tratados com metronidazol ou vancomicina. O risco de viés foi avaliado por ROBINS-I. Desfechos extraídos incluíram recorrência, sucesso clínico, colectomia e mortalidade.

**Resultados:**

Nove estudos (n=643) foram incluídos. A recorrência ocorreu em 17,4% dos pacientes tratados com metronidazol e em 8,2% daqueles tratados com vancomicina. No modelo de efeitos aleatórios, o OR foi 0,55 (IC95% 0,47-4,39; *p*=0,52; I²≈55%). Colectomia ocorreu apenas em pacientes com retocolite ulcerativa. A mortalidade total foi baixa e semelhante entre os grupos.

**Discussão:**

Observou-se tendência de menor recorrência entre pacientes tratados com vancomicina, embora sem significância estatística, sugerindo possível influência da inflamação colônica e da imunossupressão na resposta terapêutica. A predominância de colectomia em casos de retocolite ulcerativa reforça a gravidade desse subgrupo. A heterogeneidade dos estudos limita inferências definitivas, destacando a necessidade de investigações prospectivas estratificadas. Conclusão: Em pacientes com DII e ICD, a vancomicina pode ser considerada como alternativa terapêutica preferencial, particularmente em cenários de maior risco para recorrência, embora a ausência de estudos dedicados impeça conclusões definitivas. Ensaios clínicos específicos para essa população são necessários.

## INTRODUCTION

Inflammatory bowel diseases (IBD), including Crohn’s disease (CD) and ulcerative colitis (UC), are chronic immune-mediated conditions with increasing global prevalence. It is estimated that more than seven million individuals are affected worldwide, with rising incidence in both industrialized and developing countries^1^.

*Clostridioides difficile* infection (CDI) is one of the most clinically relevant infectious complications in patients with IBD, and is associated with worse outcomes such as prolonged hospitalization, higher recurrence rates, toxic megacolon, and mortality^2^ ^,^ ^3^ ^,^ ^9^. This increased vulnerability is related to factors such as chronic mucosal inflammation, intestinal dysbiosis, recurrent antibiotic exposure, immunosuppressive therapy, and frequent hospitalizations^4^.

The main antibiotics used for CDI are metronidazole, vancomycin, and fidaxomicin. However, most of the evidence supporting current therapeutic recommendations is derived from studies in the general population, without stratification by IBD status. For example, in the multicenter observational IBIS study, vancomycin was associated with higher initial clinical response (80.9%) and lower recurrence (11.7%) compared with metronidazole (72.0% and 20.0%, respectively), but patients with IBD were not analyzed separately^5^.

Fidaxomicin has been shown to reduce recurrence while preserving the intestinal microbiota and is recommended as first-line therapy for initial, non-severe CDI in international guidelines by ESCMID and IDSA/SHEA^6^ ^,^ ^7^. Recent systematic reviews reinforce the superior performance of fidaxomicin and vancomycin in immunocompromised subgroups^8^. However, there is a notable lack of prospective clinical trials specifically designed to evaluate these agents in patients with IBD.

Given these gaps, the objective of this systematic review and meta-analysis was to compare the efficacy of the most commonly used antibiotics for CDI in patients with IBD, with the goal of supporting individualized and evidence-based therapeutic decisions.

## METHODS

This systematic review was conducted in accordance with the Preferred Reporting Items for Systematic Reviews and Meta-Analyses (PRISMA) 2020 guidelines. The protocol was prospectively registered in the International Prospective Register of Systematic Reviews (PROSPERO; CRD420250491687). The objective was to compare the efficacy of the main antibiotic regimens used to treat *Clostridioides difficile* infection (CDI) in adult patients with inflammatory bowel disease (IBD).

A comprehensive search strategy was applied to MEDLINE, EMBASE, PubMed, Epistemonikos, and the Cochrane Library. The following terms and Boolean operators were used: (“Clostridioides difficile” OR “Clostridium difficile”) AND (“inflammatory bowel disease” OR “Crohn disease” OR “ulcerative colitis”) AND (metronidazole OR vancomycin OR fidaxomicin). Search strategies were adapted for each database. The search included studies published up to October 2024. Reference lists of all potentially eligible articles were also screened for additional citations. Observational studies randomized clinical trials, and gray literature (including conference abstracts with complete data) were eligible, with no language restriction.

Eligibility criteria followed the PICO framework. The population consisted of adult patients with diagnosed IBD and CDI, according to the definition used in the original studies. The intervention was antibiotic therapy for CDI. Comparators included the type of antibiotic administered (metronidazole, vancomycin, or fidaxomicin), dosage, and treatment duration. Outcomes included recurrence rates, clinical success, adverse events, mortality, and CDI-related complications.

Study screening was performed independently by two reviewers using Rayyan software. Titles and abstracts were assessed first, followed by full-text review of selected articles. Disagreements were resolved by a third reviewer. Reasons for exclusion were documented according to PRISMA requirements.

Data extracted included study characteristics (year, country, design), patient demographics (age, sex, comorbidities), IBD subtype and prior treatment, CDI diagnostic criteria, antibiotic regimen, dose, treatment duration, route of administration, and clinical outcomes (recurrence, clinical response, colectomy, mortality).

Handling of combination or sequential antibiotic therapy: patients who received metronidazole and vancomycin simultaneously were excluded from comparative efficacy analyses to avoid treatment overlap bias. For patients who initiated therapy with one antibiotic and were later switched to another, classification was based on the initial antibiotic regimen, as this reflected the primary therapeutic strategy and indication at CDI diagnosis.

Route of administration: the route of antibiotic administration (oral, intravenous, or rectal) was extracted whenever reported. However, several studies did not provide sufficient detail regarding administration routes. Therefore, this variable was described qualitatively and was not included in quantitative synthesis.

Risk of bias was assessed using the ROBINS-I tool for non-randomized studies. The certainty of evidence was evaluated using the GRADE approach.

When feasible, data were pooled using RevMan 5.4 software. Random-effects models were applied when heterogeneity was substantial. Heterogeneity was quantified using the I² statistic, with values >50% considered indicative of moderate to high heterogeneity. Results were summarized in tables and figures. Studies with high heterogeneity or insufficient data were synthesized narratively.

Although some studies reported additional outcomes such as clinical success, adverse events, length of hospital stay, ICU admission, and need for IBD therapy escalation, these variables were not consistently defined or reported across studies. Consequently, they were summarized narratively rather than pooled in meta-analysis.

## RESULTS

A total of nine studies were included, comprising 643 patients with concomitant inflammatory bowel disease (IBD) and *Clostridioides difficile* infection (CDI). The general characteristics of the patients included are summarized in Table 1. The pooled mean age was 42.6 years. Among the six studies reporting sex distribution (n=461), 183 patients (39.8%) were male and 248 (53.7%) were female, while sex was not reported for 30 individuals (6.5%).

**TABLE 1:** General Characteristics of Included Patients (n=643).

Variable	n (%) / Mean
Total studies included	9
Total patients	643
Mean age (years)	42.6
**Sex (n = 461)**	
• Male	183 (39.8%)
• Female	248 (53.7%)
• Not reported	30 (6.5%)
**IBD subtype**	
• Ulcerative colitis	Majority (predominant data)
• Crohn’s disease	Smaller proportion
**Antibiotic regimen**	
• Metronidazole	235
• Vancomycin	158
• Both (at any point)	170
**Recurrence**	
• After metronidazole	41/235 (17.4%)
• After vancomycin	13/158 (8.2%)
Colectomy (UC only)	35
**Mortality**	
• Crohn’s disease	4
• Ulcerative colitis	3

Regarding antibiotic therapy, 235 patients received metronidazole, 158 received vancomycin, and 170 were treated with both agents at different time points during their clinical course.

Recurrence was documented in 41 of 235 patients (17.4%) treated with metronidazole and in 13 of 158 patients (8.2%) treated with vancomycin.

Among clinical outcomes, 35 colectomies were reported, all of which occurred in patients with ulcerative colitis. No colectomies were described in patients with Crohn’s disease. Mortality was recorded in four patients with Crohn’s disease and in three with ulcerative colitis.

**FIGURE 1:**
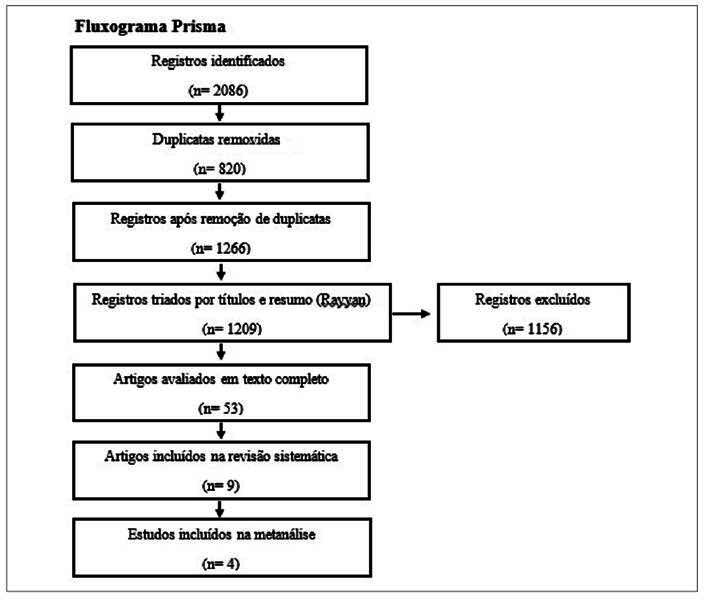
Meta-analysis of Recurrence Rates.

The meta-analysis included four observational studies (Bownik 2015; Dharbhamulla 2018; Hamid 2014; Horton 2012), totaling 214 patients, of whom 64 were treated with vancomycin and 150 with metronidazole (Figure 1). The outcome of interest was CDI recurrence following treatment.

**FIGURE 2:**
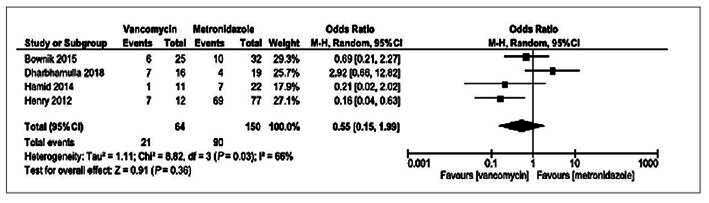
Forest plot comparing recurrence of CDI following treatment with metronidazole versus vancomycin in patients with inflammatory bowel disease. Random-effects model.

The pooled odds ratio (OR) for recurrence was 0.55 (95%CI 0.15-1.99; *P*=0.36), indicating no statistically significant difference between vancomycin and metronidazole (Figure 2). Although recurrence tended to be lower among patients receiving vancomycin, the confidence interval was wide and crossed unity, preventing definitive inference of therapeutic superiority.

Heterogeneity was moderate to high (I²=66%; *P*=0.03), suggesting variability among studies, likely related to differences in IBD severity, CDI severity, treatment regimen (initial vs rescue therapy), and recurrence definition criteria.

Additional outcomes such as clinical success, adverse events, length of hospital stay, ICU admission, and need for IBD therapy escalation were reported in some studies. However, these outcomes were not consistently defined or uniformly reported across all included studies. Due to this methodological heterogeneity, these variables could not be pooled in meta-analysis and were therefore described narratively.

## DISCUSSION

*Clostridioides difficile* infection (CDI) is a relevant complication among patients with inflammatory bowel disease (IBD), particularly those with colonic involvement and active mucosal inflammation. This subgroup is known to present a higher risk of recurrence, hospitalization, therapeutic failure, and colectomy when compared with the general population^2^ ^,^ ^3^. Intestinal dysbiosis, disruption of the mucosal barrier, and frequent exposure to corticosteroids and immunosuppressive agents contribute to increased susceptibility to infection and to worse clinical outcomes^4^ ^,,^ ^10^ ^11^.

Although fidaxomicin and vancomycin are recommended as first-line therapy in international guidelines such as those from ESCMID and IDSA/SHEA^6^ ^,^ ^7^, there is a notable lack of studies specifically evaluating the efficacy of these antibiotics in patients with IBD. This gap in the literature served as the rationale for the present systematic review and meta-analysis, which compared metronidazole and vancomycin, given the limited availability of robust data on fidaxomicin for this population.

In this review of nine observational studies involving 643 patients, we observed a trend toward lower recurrence rates with vancomycin (8.2%) compared with metronidazole (17.4%). This finding is consistent with previous reports by Horton et al.^12^, Hamid et al.^13^, and Bownik et al.^14^, which identified higher treatment failure and need for escalation when metronidazole was used as initial therapy. Gupta et al.^15^ further highlighted that diagnostic modality may influence outcomes, as PCR-only testing may overestimate colonization. Studies by Dharbhamulla et al.^16^ and Campbell et al.^17^ reinforce that antibiotic selection often reflects illness severity, which may confound direct comparison of treatment effects. Observational data from recent cohorts, including IBIS and Conrad et al.^5^, although not stratified specifically by IBD, demonstrate higher initial response and lower recurrence with vancomycin, which aligns with the trend observed in our analysis.

The predominance of ulcerative colitis (UC) among included patients and the fact that all reported colectomies occurred in UC further support the concept that this subgroup represents a clinically more severe population, characterized by extensive colonic inflammation, greater microbiota disruption, and increased risk of CDI recurrence. The mean age (~43 years) and low overall mortality observed here are consistent with previous epidemiological data^3^ ^,^ ^4^, suggesting that the studies included adequately represent the typical high-risk IBD population. The higher rate of adverse outcomes among UC patients compared with those with Crohn’s disease may be related to continuous colonic inflammation and more frequent corticosteroid exposure, both of which have been associated with CDI recurrence^11^ ^,^ ^12^.

From a pharmacological standpoint, it is plausible that vancomycin demonstrates greater clinical effectiveness in patients with significant colitis, as it remains largely within the intestinal lumen, reaching high intraluminal concentrations, whereas metronidazole depends on systemic absorption followed by mucosal diffusion - a process that may be impaired in severe colonic inflammation^8^. This mechanistic difference may account for the direction of effect observed in our analysis, even in the absence of statistical significance.

However, this review is not without limitations. Most included studies were observational in nature, introducing potential risk of selection and confounding bias. There was heterogeneity in definitions of recurrence, disease severity, diagnostic methods, and prior therapies. Few studies stratified outcomes according to IBD subtype or inflammatory activity. Additionally, the absence of data on fidaxomicin in IBD represents a relevant gap in the literature.

Despite these limitations, our findings have mea­ningful clinical implications. In patients with IBD and CDI, particularly those with extensive colonic disease, active inflammation, or elevated risk of recurrence, vancomycin may represent a reasonable therapeutic approach, although the current evidence remains limited and heterogeneous. There is also a clear need for prospective, adequately powered clinical trials comparing fidaxomicin and vancomycin directly in IBD-specific subgroups stratified by disease severity, inflammatory biomarkers, and immunosuppressive therapy.

Another important limitation is the lack of standardized reporting of clinical success, adverse events, and treatment-related complications across studies. Although some articles described these outcomes, definitions and measurement methods varied considerably, preventing quantitative synthesis. This heterogeneity reflects a broader limitation of the existing literature rather than a methodological weakness of the present review.

Overall, our findings reinforce that patients with IBD should not be managed using the same therapeutic framework applied to the general population with CDI. The inflammatory environment, degree of mucosal injury, and frequent immunosuppression fundamentally alter disease presentation and therapeutic response. The absence of dedicated clinical trials in this population remains a critical limitation, and future research is essential to guide individualized, evidence-based management strategies.

## CONCLUSION

In this systematic review and meta-analysis, we evaluated the efficacy of different antibiotic therapies for *Clostridioides difficile* infection (CDI) in patients with inflammatory bowel disease (IBD). Our findings showed a trend toward lower recurrence rates among patients treated with vancomycin compared with metronidazole; however, this difference did not reach statistical significance.

Despite the heterogeneity across included studies and the limited availability of stratified data, the results are consistent with emerging clinical observations suggesting that vancomycin may be associated with lower recurrence in IBD patients, particularly those with active colitis, extensive disease, or increased risk for recurrence.

These findings emphasize the importance of individualized therapeutic decision-making, considering the clinical context of IBD, infection severity, and the potential for treatment failure. The absence of dedicated clinical trials evaluating fidaxomicin and vancomycin specifically in IBD highlights a significant gap in the literature.

Future prospective and randomized studies, stratified by disease phenotype and inflammatory burden, are necessary to clarify treatment effects and provide stronger evidence to guide management in this high-risk population.

## Data Availability

Data availability statement: Data-available-upon-request
